# The Legal Doctrine of Responsibility in Cases of Insanity, Connected with Alleged Criminal Acts

**Published:** 1858-04-01

**Authors:** Forbes Winslow


### ART. II.—THE LEGAL DOCTRINE OF RESPONSIBILITY IN CASES OF INSANITY, CONNECTED WITH ALLEGED CRIMINAL ACTS.

BY FORBES WINSLOW, M.D., D.C.L.

*Read at the Juridical Society, December 14th,* 1857.

I WILL, without any prefatory remarks, and with great submission to those whom I have the honour to address, endeavour to direct the attention of the Society to the following salient and relevant questions connected with the important subject selected for discussion.

I will consider *seriatim:*—

1st. The nature of insanity in its medico-legal relations.

2nd. The legal doctrine of responsibility in connexion with insanity associated with alleged criminal acts.

3rd. The doctrine of partial insanity, or monomania.

4th. The existence of homicidal insanity and insane irresistible impulses.

5th. Anomalous or mixed cases of mental disorder, involving the question of modified responsibility and the propriety of punishment.

There is no fallacy more generally entertained by those who have had but limited opportunities of studying or becoming practically acquainted with the phenomena of insanity, than that, in the great majority of cases, the disease consists, in its elementary and *essential features*, in a disorder of the *intellectual*, as contra-distinguished from a derangement of the *moral* faculties of the mind; that the intellect is in a condition of *aberration;* that the *ideas* are *perverted;* that the senses convey illusory images to the sensorium; that the perceptions are false, the mind being invariably under the dominion of some creation of the distempered fancy; in other words, that delusions or hallucinations are always present in every case of fully developed insanity.

This is the popular and, I may add, the generally received notion of mental derangement.

This mistaken view of the nature of insanity has, I believe, led to much discrepancy and conflict of opinion in our courts of law respecting the legal question of responsibility in connexion with certain cases of imputed alienation of mind.

In all the great criminal trials involving a consideration of this question, the judges have almost invariably laid a stress on the presence or absence of *delusion;* associating it, however, with the question, "Is the person whose mind is said to be insane, capable of distinguishing right from wrong?"

In the case "Bainbridge *v.* Bainbridge," Lord Campbell admitted that insanity might exist without delusion. I have no doubt other judges, if they have not propounded literally the same doctrine, have practically acknowledged its truth, by sanctioning the acquittal of prisoners on the ground of insanity unassociated with any obvious delusion or affection of the reasoning powers.

It is difficult for an inexperienced person to realise the great medical truth, that disease of the mind, and disease of a serious character, may exist *without any appreciable aberration of the ideas, or apparent impairment of what are termed the intellectual powers.* I do not refer to conditions of morbid mental exaltation, often dependent upon a transitory congestion of the blood-vessels on the surface of the brain, or to that mental depression so frequently consequent upon an obstruction to the free circulation of the blood through the heart, or even to the extravagance of thought and conduct exhibited in many cases of unrecognised insanity; but to positive creations of the morbid fancy, to delusive images leading the person to believe that to exist which no sane person would believe to exist, and which, in reality, has no existence apart from himself and his distempered imagination.

In the majority of cases, the premonitory stage of insanity is evidenced by some palpable disorder of the affections, temper, propensities, moral sense, character, and conduct of the individual. This may exist for a long period before any positive aberration of the ideas is recognised.

It is unusual for delusions to exist in the early stage of mental derangement. The poison of insanity, if I may use the term, seizes, in the first instance, hold of the moral powers of the mind, and the disease often runs its course without obviously deranging the ideas, perceptions, or apparently impairing the integrity of the intellectual operations. Men talk coherently, and often with great shrewdness and sagacity, and they occasionally write rationally whilst in an indisputable condition of mental aberration of such a kind and degree as clearly to absolve them from all legal responsibility.

Although, as Dr. Prichard justly observes, "the intellectual faculties in every case of well-marked insanity are more or less involved;" and this will be apparent when I address myself to the question of partial insanity and the metaphysical doctrine of the indivisibility of the mind and unity of the consciousness; still he allows that "in reality the *moral character* is more affected than the *understanding*." In other words, he maintains that the salient, prominent, characteristic, and diagnostic symptoms of insanity are not to be sought for in those faculties of the mind by which (to speak with metaphysical exactness) *we appreciate the perception of relation;* but in those states and conditions of the intellect more immediately associated with the moral sense, the *affective* or *motive* faculties, the *passions, affections,* and *appetites.* As a general rule, insanity implicates those powers of the mind which are supposed to regulate the actions and conduct. The intellectual as well as the moral faculties (from the nature of the constitution of the human mind) are in all cases of insanity to a certain degree disordered; but the affection of the *reason,* the *judgment,* and *reflection,* does not in many cases stand out in bold and prominent relief, so as to constitute well-marked legal or medical diagnostic indications of the actual state of the mind when affected by disease. If this be a true theory of insanity, it will be apparent that, in estimating the actual condition of the mind in connexion with the question of legal responsibility, we must not confine our attention to the question, whether the *ideas* are perverted or in a state of positive aberration or derangement; whether the senses are under the influence of subjective or objective morbid psychical phenomena, in the form of hallucinations or illusions; but the important point for consideration should be, what is the state of the *affective* or *motive* powers? what is the condition of the *volition?* and to what degree has the mental disease destroyed the healthy power of self-control over the thoughts and actions? If delusions are present—if hallucinations and illusions can be detected, the diagnosis is greatly simplified; but although delusions and aberration of the ideas often exist, *they must not be viewed as the essential or the exclusive diagnostic symptoms of a diseased and irresponsible mind.* If a man is said to be insane, the immediate question is, what are his delusions? If evidence is given of insanity in a court of justice, the same question is often put to the witness.

I do not complain of this course of interrogation; but I argue, *that by always searching for delusions and hallucinations, or some form of aberration and derangement of the mental operations,* we are diverted from the legitimate and philosophical course of inquiry, and a case of insanity, and insanity clearly inducing a state of criminal irresponsibility, eludes our observation.

I proceed to consider, secondly, "*the legal doctrines of irresponsibility in connexion with alleged criminal acts.*"

This necessarily compels me to direct the attention of the Society to the lucid, logical, and able paper on Insanity in its Legal Relations, read by Mr. Fitzjames Stephen in the month of June, 1855, and since published in the Transactions of this Society. This gentleman, when speaking of legal tests of insanity, argues that medical men have no right to charge the judges with having propounded a fallacious "*test*" of insanity, or with inconsistency in excusing the insane on the one hand, whilst on the other they apply a criterion bringing nearly all those who are insane within the range of the law. The judges, it is said, have laid down no test of insanity whatever—that they have been most scrupulously cautious and careful in committing themselves to anything like a test of insanity. Instead of so doing, Mr. Stephen affirms they have merely laid down tests of *responsibility*, or, more strictly speaking, have specified facts from which, when juries have found them, judges are to infer malice; but it is no part of their duty to say how far particular diseases affect the relation of persons to such tests; that, in the language of Mr. Justice Maule, is a question not of law but of physiology, and one not of that obvious nature to be inferred without proof. The tests of insanity, as I conceive, propounded in our courts of law, are as follows, viz.:—

1st. That of the presence of delusion.

2nd. Of delusion directly associated with the criminal act.

3rd. A capability of distinguishing between what is lawful and unlawful—the capacity of knowing right from wrong, good from evil.

All these legal *criteria* of insanity necessarily involve in their elucidation the question of responsibility.

It is true, as Mr. Stephen argues, that, stripped of all technicalities, the transaction, as between the criminal arraigned for the crime and the prosecution, stands thus:—

The prosecutor says, "I charge this man with having voluntarily and wickedly killed A. B." The prisoner replies, "I did kill him, but not voluntarily or wickedly; for I was compelled by the involuntary action of my muscles, and exercised no volition in the matter; or, I was prevented by disease from distinguishing good from evil, and, therefore, could not act wickedly." But does not this trial of the question, whether the accused, by reason of his incapacity, could not act feloniously, unavoidably raise the questions, what is the nature of this incapacity? how is it manifested? what are its symptoms? is it partial or general incapacity? is it associated or dissociated with delusions? does the mental disorder destroy the prisoner's power of distinguishing between what is "lawful and unlawful," "good and evil," "right and wrong?" Am I not justified in maintaining, without arguing the question in a "Nisi prius" spirit, that legal tests of insanity connected with alleged criminal acts have at various times been propounded from the bench for the guidance of juries?

Dr. Johnson defines the word "test," as being "that with which anything is compared in order to prove its genuineness."

It is true, as Mr. Justice Maule says in the passage quoted by Mr. Stephen, that the questions submitted to the jury are those questions of fact which are raised on the record. In a criminal trial the question commonly is, whether the accused be Guilty or Not Guilty?

"The jury are to inquire into nothing which is not in issue. They are impannelled to decide certain questions of fact in the negative or affirmative, and nothing is admissible in evidence unless it tends to enable them to answer these questions, or some of them.

"The questions are raised by the prosecutor and the prisoner—the prosecutor affirming certain facts respecting the prisoner, and the prisoner either confessing or denying them, or alleging some reason why he should neither confess nor deny. Such denial, confession, or allegation, is the prisoner's plea; and if it raises a question, asserted on one side and denied on the other, the jury are to decide it. First, then, madness is not a plea.

The prisoner does not plead it as he would plead a pardon under the Great Seal, a former acquittal or conviction, or as he would plead to the jurisdiction. He gives it in evidence under the plea of Not Guilty. So that the very form of the proceedings implies, that, in order to entitle him to an acquittal, the prisoner must not only show that he is mad, but that he is *thereby* not guilty. In more technical language, his madness must be such as to enable him to traverse some one or more of the material averments of the indictment."

Madness may not, to speak with technical accuracy, be the "plea," as Mr. Justice Maule avers; but are not the jury guided in their decision as to the acquittal of the prisoner on the ground of insanity by the judge's exposition of the legal doctrine of insanity in relation to crime? Does he not instruct the jury that "partial insanity" will not acquit the prisoner? That the existence of a delusion, partial in its character, will not exonerate him from responsibility? That if the prisoner was labouring under the idea or delusion that he was "redressing a supposed grievance," and that under "the impression of obtaining some public or private benefit" he committed the crime, he is equally liable to punishment?

Surely these instructions, propositions, doctrines, or theories may, without an abuse of language, be also termed *tests* of insanity and responsibility, in relation to certain alleged morbid conditions of thought and conduct. The law has a certain preconceived standard of criminality. The mind of the alleged criminal must be in a condition to act voluntarily, of free will, and with malice. He must, to use the language of Foster, as quoted by Mr. Stephen, be capable of committing an action flowing from a wicked and corrupt motive; he must be in a condition to act *malo animo malâ conscientiâ.*

"If a man," says Foster, "has either no motive at all, or no power of discerning what motives are wicked, and what are not —in more popular language, if he cannot discern good from evil, he cannot be said to act maliciously in the legal sense of the word; and if he can show, by reason of any disease, he is wholly unable to distinguish between good and evil, he has rebutted the presumption of malice."

Let us for a moment apply Dr. Johnson's definition of the word "test" to this lucid exposition of the principles of the criminal law, and how does it affect the question at issue?

A standard of criminal responsibility is erected; in other words, certain well-defined principles of criminal responsibility are enunciated. A culprit is indicted for murder; he pleads Not Guilty, on the ground that he was incapable of acting *voluntarily, maliciously,* and of *free will,* on account of his mental infirmity destroying his power of distinguishing between good and evil, right and wrong. This condition of alleged and imputed disorder of mind is then reviewed by the Court, and its "genuineness" tested by "comparing" it with those mental states of admitted legal responsibility in which the criminal is capable of acting *malo animo malâ conscientiâ.*

What are the doctrines of criminal responsibility in cases of alleged insanity, as propounded authoritatively in our courts of law? I will not refer in detail to the conflicting *criteria* of responsibility which have at different periods been laid down by the bench. (For the existence of such discrepancy of opinion was candidly admitted by Lord Campbell, in the House of Lords, when he said, "He had looked into all the cases that had occurred since Arnold's trial, in 1723, and to the direction of the judges in the case of Lord Ferrers, Bellingham, Oxford, Francis, and M'Naughton, and he must be allowed to say that there was a wide difference of opinion both in the *meaning* and in the *words* of their description of the law.") The principle of law as expounded in 1843, by the judges in the House of Lords, appears to me (without quoting the decision at length) to be embraced in the following propositions:—

1st. A person labouring under partial delusions only, and who is not in other respects insane, notwithstanding he commits a crime under the influence of the insane delusion that he is redressing or revenging some supposed grievance or injury, or producing some public benefit, is liable to punishment if he knew at the time of committing such crime that he was acting contrary to the law of the land.

2nd. To establish a defence on the ground of insanity, it must be clearly proved that at the time of the committing of the act the party accused was labouring under such a defect of reason from disease of the mind as not to know the nature and quality of the act he was doing, or, if he did know it, that he did not know he was doing what was wrong.

3rd. If a person under a partial delusion only, and not in other respects insane, commits an offence in consequence thereof, he is to be considered in the same situation as to responsibility as if the facts in respect to which the delusion exists were real.

These rules of law in relation to offences committed in an alleged condition of insanity, suggest for consideration—

1st. The doctrine of partial delusions in their legal relation to crimes committed by persons in other respects insane, under an insane idea of redressing a real injury, or revenging some supposed grievance.

2nd. The legal doctrine of partial insanity.

3rd. The knowledge of right and wrong viewed as conclusive evidence of responsibility in cases of imputed insanity.

Before discussing the question of "partial insanity," I would premise that the rule of law by which persons are held legally responsible for actions committed whilst under the dominion of a delusion, provided the person imagined that he was redressing a *supposed* grievance, or under the impression of obtaining some public or private benefit, was propounded by Lord Erskine in his celebrated speech in defence of Hadfield. He there declared, "That when a madman commits a crime under the influence of an impression which is entirely visionary, and purely the hallucination of insanity, he is not the object of punishment; but that though he may have shown insanity in other things, he is liable to punishment if the impression under which he acted was true, and the human passion arising out of it was directed to its proper object." He illustrates this principle by contrasting the case of Hadfield with that of Lord Ferrers. Hadfield laboured under the delusion that the end of the world was at hand, and that on the death of the king, the Messiah would immediately appear on earth, and the reign of the Millennium begin.* Lord Ferrers, after showing various indications of insanity, murdered a man against whom he was known to harbour deep-rooted resentment on account of *real* transactions in which that individual had rendered himself obnoxious to him. The former, therefore, is considered as an example of the pure hallucination of insanity; the latter, as one of human passion founded on real events, and directed to its proper object. Hadfield was accordingly acquitted, but Lord Ferrers was convicted of murder, and executed.

It will be for us to discuss whether it is consistent with an enlightened jurisprudence, and a philosophic view of insanity, to consider that a man in an insane state of mind should be held amenable to the punishment of death, *because his delusion is to a degree based upon actual circumstances, and because there is in his conduct evidence of his having been under the influence of passion apparently rationally and sanely directed?*

In considering this section of our subject, it is essential that we should fully appreciate the fact, that it is one of the well-known characteristics of insanity for persons to labour under

* Although very insane, Hadfield exhibited great acuteness, coolness, and self-possession, common features in cases even of dangerous insanity. It is stated that when standing at the pit-door of the theatre, waiting for admission, the people around pressed and crowded inconveniently upon him, when a young woman, putting her hand on his shoulder, said, "Sir, you are hurting me; the handle of your umbrella is running into my bosom." "I could not," he added, "help smiling at the time, for the handle of what she supposed my umbrella was the handle of my pistol, which I held concealed within my coat under my arm."

delusions connected with, and originating in, actual circumstances. This is one of the common features of insanity, the mental disorder exhibiting itself in a morbid and false view of the actual objects of sense, and a diseased and exaggerated estimate of the daily occurrences of life.

A man in a state of incipient or advanced insanity notices a person paying more than (he considers) the ordinary, legitimate, and conventional attention to his wife. A case can easily be conceived in which a man may, in this respect, unintentionally slightly overstep the line of prudence and propriety. The fact is observed by the *quasi* suspicious madman, and made the subject of deep thought and meditation, until the mind, being up to this period only in an incipient condition of lunacy, yields to the morbid mental suggestion that his wife has been actually unfaithful, and that the man who has been seen in apparent familiar converse with her is her seducer. Thus may a delusion —a dangerous, an insane delusion—a delusion based upon a distorted, perverted, irrational, and insane view of *actual circumstances,* originate and impel the person to destroy human life. I will imagine a case like M'Naughton's. A person is under a delusion that he is the victim of a conspiracy. His insanity may be somewhat general in its development—his delusions not, in the first instance, attaching to any one particular individual, or, in legal phraseology, his insanity is not yet "partial" in its manifestation.

It is possible that a man in such a state of mind may have some trifling claim upon the Government for either insignificant services rendered to a Cabinet Minister, or on account of property sacrificed in defence of the Crown in one of our colonial possessions. He writes and demands compensation—extravagant compensation—for a questionable service rendered, and a still more doubtful injury sustained. He is told that his claims are all illusory. This disappointment preys upon his mind, until his bodily health becoming vitiated, and his mind palpably disordered, the idea of the wrong inflicted becomes *a fixed, false, and delusive impression,* exercising a tyrannical and autocratic sway over his passions and conduct. His disordered fancy fixes upon one of the Government officials—it may be one of the clerks of the office with whose chief he has been in correspondence—and under the dominion of this phantom of his imagination, that he has a *bonâ fide* claim which will not be recognised, and rights which are unjustly ignored, he revenges himself by taking his life! Alter the circumstances, and it constitutes a type of case frequently coming under the observation of persons conversant with insanity. Many of the delusions of the insane may thus be traced to actual existing circumstances.

A merchant becomes to a degree affected in his pecuniary circumstances; he has sustained a trifling loss of property. This disturbs his thoughts, interferes with his regular sleep, and eventually damages the general health. His mind ultimately succumbs to the brain disorder, and symptoms of unmistakeable insanity appear. He is under a delusion that he is reduced to a state of abject poverty, declares that he is not worth a farthing, and asserts that he and his family must go to the workhouse. It is useless to reason with a man so insane. A clear statement of his affairs is laid before him, he listens heedlessly to the representations of his kind relations and friends, and appears to examine his banker's book with care, but nothing dissipates the delusion; there it remains a fixed, permanent impression of hallucination, until death puts a period to his unhappy life. This is a case of insanity springing out of actual circumstances; the disease of the mind evidencing itself in a false, perverted, insane, and irrational estimate of events that have in reality taken place. In many of these cases the mind is in an incipient state of disorder before the occurrence of the shock, and the palpable demonstration of derangement which afterwards exhibits itself is only a continuation of a previously existing state of mental alienation; but this does not in the slightest degree affect the principle for which I am contending,—that many commit offences against the law in an irresponsible state of insane mind, who are considered accountable agents and amenable to punishment, because they act under a delusion that they are redressing a supposed grievance; and, having some slight justification for their impressions, proceed and conduct themselves as a man in sane and healthy possession of his reason would under similar circumstances. The law assumes that persons in an irresponsible state of insanity do not redress injuries like sane men; that they are oblivious to all feelings of revenge and resentment; that they are incapable of feeling the

> "Whips and scorns of time,

> The oppressor's wrong—the proud man's contumely."

So much for the rule of law laid down for the guidance of those into whose hands are entrusted the administration of justice—viz., that "notwithstanding the party (the insane party) committed a wrong act while labouring under the idea (delusion, I presume) that he was redressing a supposed grievance or injury (a fanciful and imaginary grievance and injury), or under the impression (hallucination) of obtaining some public or private benefit, he is liable to punishment." I maintain that this is an erroneous doctrine of responsibility in cases of alleged insanity, and an unsafe principle of law; because it is based upon false views of the true characteristics and phenomena of mental alienation. I am not now addressing myself to the consideration of incipient forms of disturbed mind, to *pseudo* states or phases of insanity, or to certain abnormal deviations from mental health, not amounting to derangement of mind; but to clearly, positively, and obviously developed insanity, associated with palpable and appreciable delusions or hallucinations. With reference to the legal doctrine of right and wrong as applied to cases of alleged insanity, I suggest no metaphysical objection. I use the words in their admitted and recognised legal acceptation. The word *wrong*, as Mr. Stephen observes, is "that which the *law* and not that which the *prisoner* considers wrong."

It is questionable whether the English language could produce two words so incapable of uniformity of construction as those of right and wrong when applied to criminal cases of insanity.

If the doctrine of right and wrong be admitted as a legal test, and acted upon as a principle of law, would it not (owing to the essential difference in the character of the cases of insanity to which it would be applied) be partial, restricted, and circumscribed in its operation? There are, undoubtedly, cases of insanity which come within the range of this test; but in many forms of mental disorder associated with an irresponsible condition of mind, the doctrine of right and wrong could not with justice or safety be relied upon.

If it be a fact that there are a large number of insane persons confined as lunatics, in whom this power of distinguishing between right and wrong, lawful and unlawful, good and evil, remains apparently intact, then, I ask, is it a safe standard of responsibility—a just principle of law?

I say advisedly, "apparently intact." A lunatic may have one or two prominent delusions, and in this state of disordered intellect retain the power of conversing coherently, rationally, and even with brilliancy, upon many subjects connected with science, literature, and the fine arts. He may even be competent to make a testamentary disposition of his property, and to transact ordinary matters of business with unusual shrewdness and a keen regard to self-interest; the fact of his brain being in a morbid state, in a condition of exalted function, may develop an amount of intelligence, acuteness, and sagacity he never exhibited previously to the attack of mental disorder. Hence the extreme cunning, cleverness, and design often exhibited by persons palpably insane. Men in a state of insanity become orators and poets, who previously to their illness were entirely ignorant of tropes, and innocent of ever having penned a stanza. But we must be careful not to confound such conditions of *morbid exal-*

*tation of thought and intelligence* with those complex operations of the mind, involved in the consideration of the question of right and wrong, under circumstances the most painful and trying that can occur to a human individual. In other words, I argue, that the capacity to draw nice distinctions between right and wrong—the power of correctly estimating the relation between a suggested line of action, and its penal consequences,—the ability to appreciate in a healthy manner the moral and legal principles laid down for the conduct of society, and the safety and protection of human life, are not to be confounded with an apparent lighting up of the intelligence so often witnessed in certain morbid conditions of the brain, disordering the operations of the mind. We are not justified in inferring, because the alleged lunatic exhibits more than the usual degree of cleverness, cunning, and sagacity, that therefore he is in a condition of intellect to weigh nicely and accurately (when impelled, in an insane state of mind, to commit an act of violence upon a fellow-creature who had subjected him to a slight provocation) the questions—Am I doing what is right? what is lawful? what is good? am I about to act in disobedience to human and Divine laws?

Dr. Ray has placed this question in a clear and forcible light:—

"The first result, therefore, to which the doctrine leads, is, that no man can ever successfully plead insanity in defence of crime, because it can be said of no one, who would have occasion for such a defence, that he was unable in any case to distinguish right from wrong. To show the full merits of the question, however, it is necessary to examine more particularly how far this moral sentiment is affected by, and what relation it bears to, insanity. By that partial possession of the reasoning powers, which has been spoken of as enjoyed by maniacs generally, is meant to be implied the undiminished power of the mind to contemplate some objects or ideas in their customary relations, among which are those pertaining to their right or wrong, their good or evil, tendency; and it must comprise the whole of these relations, else the individual is not sane on these points. A person may regard his child with the feelings natural to the paternal bosom, at the very moment he believes himself commanded by a voice from heaven to sacrifice this child, in order to secure its eternal happiness, than which, of course, he could not accomplish a greater good. The conviction of a maniac's soundness of mind, on certain subjects, is based in part on the moral aspect in which he views those subjects; for it would be folly to consider a person rational in reference to his parents and children, while he labours under an idea that it would be doing God's service to kill them,—though he may talk rationally of their characters, dispositions, and habits of life, their chances of success in their occupations, their past circumstances, and of the feelings of affection which he has always cherished towards them.

"Before, therefore, an individual can be accounted sane on a particular subject, it must appear that he regards it correctly, in all its relations to right and wrong. The slightest acquaintance with the insane will convince any one of the truth of this position. In no school of logic, in no assembly of the just, can we listen to closer and shrewder argumentation, to warmer exhortations to duty, to more glowing descriptions of the beauty of virtue, or more indignant denunciations of evil-doing, than in the hospitals and asylums for the insane. And yet many of these very people make no secret of entertaining notions utterly subversive of all moral propriety; and, perhaps, are only waiting a favourable opportunity to execute some project of wild and cruel violence. The purest minds cannot express greater horror and loathing of various crimes than madmen often do, and from precisely the same causes. Their abstract conceptions of crime, not being perverted by the influence of disease, present its hideous outlines as strongly defined as they ever were in the healthiest condition; and the disapprobation they express at the sight arises from sincere and honest convictions. The *particular* criminal act, however, becomes divorced in their minds from its relations to crime in the *abstract;* and, being regarded only in connexion with some favourite object which it may help to obtain, and which they see no reason to refrain from pursuing, is viewed, in fact, as of a highly laudable and meritorious nature. Herein, then, consists their insanity; not in preferring vice to virtue, in applauding crime and ridiculing justice, but in being unable to discern the essential identity of nature between a particular crime and all other crimes, whereby they are led to approve what in general terms they have already condemned."

Mr. Stephen, although he argues in favour of this doctrine of criminal responsibility, appears to consider that the question might with safety be modified. As suggested by this gentleman, the case would be thus put to the jury:—"Was the prisoner prevented by mental disease from appreciating the reasons for which the law has forbidden the crime of which he is accused, or from applying them to his own case?" I would add to these questions these words:—and was he able to exercise a healthy volition in the matter?—had his mental disease destroyed his powers of free-will *quoad* the crime of which he stands accused? A paralytic may know that, under certain conditions of danger, the only safety is in flight. He is conscious of the fact, but his *motor* power is gone. It is so with many lunatics; they know what is right, and bitterly lament their sad loss of volitional power, as well as their incapacity to act in obedience to their notions of what is right and just. This will be more apparent when I address myself to the consideration of the subject of Homicidal Insanity.

I proceed next in order to the question of Partial Insanity. Lord Hale says:—

"There is a partial insanity and a total insanity of mind. The former is either in respect of things *quoad hoc vel illud insanire;* some persons that have a competent use of reason in respect of some subjects are yet under a particular *dementia* in respect of some particular discourses, subjects, or applications; or else it is partial in respect of degrees; and this is the condition of very many, especially melancholy persons, who for the most part discover their defect in excessive fears and griefs, and yet are not wholly destitute of reason; and this partial insanity seems not to excuse them in the committing of any offence for its matter capital; for, doubtless, most persons that are felons of themselves, and others, are under a degree of partial insanity when they commit these offences. It is very difficult to define the invisible line that divides perfect and partial insanity; but it must rest upon circumstances duly to be weighed and considered both by judge and jury; lest on the one side there be a kind of inhumanity towards the defects of human nature; or on the other side, too great an indulgence given to great crimes."

And the same learned judge adds, "that the best measure is this—such a person as is labouring under melancholy distempers hath yet ordinarily as great understanding as ordinarily a child of fourteen years hath, is such a person as may be guilty of treason or felony?"*

"The term partial insanity," says Collinson, "imports that a person is insane on one or more particular subjects only, and sane in other respects." Lord Lyndhurst, who takes a more enlarged view of the subject of partial insanity, thus defines it:—He says, "the mind is not unsound on one point only and sound in all other respects, but this unsoundness manifests itself principally with reference to some particular object or person." But other authorities use the term in a restricted *sense*, synonymously with that type of mental disease called "monomania," or delusion upon one prominent topic or directed to one particular person, the mind being sound on all other subjects. Accepting this as the legal signification of the term, I ask—Is there a condition of mind which can be correctly designated as partial insanity or monomania?

Considering the matter metaphysically, I would observe, that we cannot disentangle and separate the intellectual faculties as we can the threads of a skein of silk, and say this faculty of the mind operates by itself, and that faculty is independent of the other powers of the intellect, and another state of the mind is isolated from all other conditions of mental manifestation. This is contrary to the first and elementary principles of the science of mental philosophy.

Sir William Hamilton remarks:—

"It should ever be remembered that the various mental faculties

* Hale's P.C. 30.

are only possible in and through each other; and our psychological analyses do not suppose any real distinction of the operations which we discriminate by different names. Thought and volition can no more be exerted apart than the sides and angles of a square can exist separately from each other."

Whatever classification of the faculties of the mind the metaphysical philosopher may adopt, whether it be the general division of the mind made by the ancients into the powers of the understanding, and the powers of the will, these faculties never were presumed to be so many distinct and separate entities, capable of acting independently of each other; but they have always been regarded as links of the same chain, elements of the same intellectual system. The idea of disease being restricted to one faculty of the mind, and uninfluencing other powers of the intellect, is opposed to the metaphysical theory of the unity of the consciousness. If I may quote Holy Scripture in illustration of this subject, I would refer to a portion of the 12th chapter of the 1st Book of Corinthians, in which, speaking of the indivisibility of the body, and unity of physical operation, this great principle is lucidly enunciated:—"If the foot shall say, Because I am not the hand, I am not of the body; is it therefore not of the body? And if the ear shall say, Because I am not the eye, I am not of the body; is it therefore not of the body? If the whole body *were* an eye, where *were* the hearing? If the whole *were* hearing, where *were* the smelling? But now hath God set the members every one of them in the body, as it hath pleased him. And if they were all one member, where *were* the body? But now *are they* many members, yet but one body. And the eye cannot say unto the hand, I have no need of thee; nor again the head to the feet, I have no need of you. And whether one member suffer, all the members suffer with it; or one member be honoured, all the members rejoice with it."

Apply this principle to the operations of the mind, and inquire whether the memory can say to the attention, I have no connexion with you; whether the reflective powers can say to the judgment and reason, I am independent of your co-operation; whether the will can stand aloof from the imagination; and, to speak more generally, whether the active can exist apart from the passive powers of the mind; whether the moral faculties can exercise an independent sovereignty and dominion without influencing and calling into active operation the intellectual portion of man's complex organization?

Is there not a mysterious, inscrutable, and inexplicable *oneness* in the constitution of the human mind, defying all attempts at an accurate and minute classification and separation of its powers? If such a state of mutual dependence, action, and union obtains between various states of mind (I will not use the arbitrary terms "faculty" or "power") in a condition of health, *à fortiori* how impossible is it to disjoint, separate, and individualize the mental faculties when under the influence of disease? Can we draw the line of demarcation between a diseased and healthy condition of the delicate structure of the vesicular neurine of the brain? Is it not obviously impossible for the most experienced anatomist to say, This is the territory which separates the morbid from the healthy portion of the brain? or for the physician to assert such an extent of disorder of the mind is consistent with safety and responsibility, but beyond the boundary, danger and irresponsibility commence?

When speaking of partial insanity, as an accepted legal phase and type of mental derangement, Lord Brougham remarks:—

"We must keep always in view that which the inaccuracy of ordinary language inclines us to forget, that the mind is one and indivisible; that when we speak of its different powers or faculties—as memory, imagination, consciousness—we speak metaphorically, likening the mind to the body, as if it had members or compartments; whereas, in all accuracy of speech, we mean to speak of the mind acting variously—that is, remembering, fancying, reflecting; the same mind, in all these operations, being the agent. We therefore cannot, in any correctness of language, speak of general or partial insanity; but we may, most accurately, speak of the mind exerting itself in consciousness without cloud or imperfection, but being morbid when it fancies: and so its owner may have a diseased imagination; or the imagination may not be diseased, and yet the memory may be impaired, and the owner be said to have lost his memory. In these cases, we do not mean that the mind has one faculty—as consciousness—sound, while another—as memory or imagination—is diseased; but that the mind is sound when reflecting on its own operations, and diseased when exercising the combination termed imagining, or casting the retrospect called recollection. This view of the subject, though apparently simple, and almost too unquestionable to require, or even justify, a formal statement, is of considerable importance when we come to examine cases of what are called, incorrectly, partial insanity, which would be better described by the phrase 'insanity' or 'unsoundness,' always existing, though only occasionally manifest."

But, apart altogether from the metaphysical objection to the theory, let us for a moment consider whether such a form of disease as partial insanity or monomania comes under the observation of the practical physician. There are, undoubtedly, forms of insanity in which there is an unhealthy *predominance* and exaltation given to particular mental *impressions* or *delusions*; that certain states of morbid thought and feeling stand out in bold and prominent relief, giving, as it were, a character or type to the mental disease; *but I never yet saw a case of alienation of mind in which the delusion or hallucination was in reality confined to one or two ideas, those ideas exercising no influence over the conduct of the person, and not implicating, to a certain degree, the other faculties of the mind.* It is impossible to circumscribe the operation of morbid conditions of thought, or to draw a line of demarcation between those states of mind that are clearly under the influence of disease, and those operations or faculties of the intellect that remain apparently unaffected. A man believes himself to be our Saviour, or Mahomet the prophet. Apparently the man's mind is sound upon all other points; but within what limits can we confine and restrain the influence of so serious a delusion?

A slight accession of bodily disease, a severe attack of indigestion, congestion of the liver, or a torpid state of the bowels, may make all the difference between security and safety in such a case. A person labouring under the dominion of one palpable, insane delusion or hallucination (I am now using the term delusion in its strictly medical acceptation), ought not to be treated *quoad* the question of criminality as a sane and rational man. But let me for a minute revert to the question as to the existence of partial insanity, or monomania. Foville, a French physician of great celebrity, who had for many years the medical charge of the Charenton Lunatic Asylum near Paris, when speaking of monomania, observes:—

"Monomania consists in a delirium, partial and circumscribed to a small number of objects. Monomania, in its most simple condition, is excessively rare: the number of patients who only rave on one subject is *infinitely small* compared to the number of those who are *called* monomaniacs. Under this head are often confounded all those who have some habitual dominant idea. I have only seen two cases which rigorously merit the name, and these two even were affected from time to time with more extended delirium."

He again remarks:—

"Let any one examine the hospitals of Paris, of Bicêtre, of Charenton, and he will see that, amongst the thousands of insane, there is scarcely one true monomaniac, *perhaps not one.* Insanity attacks principally, at one time the intellectual, at another the moral or affective faculties; and, again, the sensations and movements. Each of these may be more or less affected than the others; and so, when the intellect, *without being unaffected,* is less deeply involved than the other faculties, we fall into the error of considering it sound, and calling these monomaniacs. Indeed, it seems to me as though the descriptions of monomania had been written *upon the word*, and not from nature; that is to say, that writers have described what *might* merit the title of monomania, but of which they can find no instance in practice."

Moreau, also a great authority in France, says:—

"It is impossible to admit that the intellectual faculties can be modified in a partial manner. In the slightest as well as the most severe forms of insanity, there is necessarily a complete metamorphosis —a radical and absolute transformation of all the mental powers of the ONE. In other words, we are insane or we are not insane; we cannot be half deranged or three-quarters, full face or profile."

Baillarger, an eminent French psychological physician, adopts the same view of the question, and maintains that the alleged monomaniacal idea is more frequently *predominant* than *exclusive.* If we look to Germany, we find the first psychological authority of that country, Damerow, declaring that "he never knew a case of the disease of the mind called monomania, in which there was not a fundamental, general psychical disorder."

When addressing himself to the subject of monomania, Mr. Stephen remarks, that "monomaniacs are capable of acting quite rationally upon a variety of subjects except those which they connect with their delusions." Apparently, such is the fact. If a person be under a delusion—an insane delusion—that he is a pauper, he having at the time large landed possessions, as well as a considerable balance at his banker's; if that be his hallucination, how can it be safely predicated that all his thoughts and feeling may not be materially tinctured and influenced by his morbid state of mind? He may be able to solve a problem in mathematics—he may have the power of writing a consistent letter on business to his solicitor—and on some subjects, involving an exercise of the intellectual faculties, his mind may appear sound; but on matters which are likely to call into play his passions, feelings, and affections, or to tax severely the emotions, his power of acting sanely and responsibly may be altogether destroyed. I maintain that it is not right to place a man whose mind is palpably deranged, even although to a partial extent (adopting the legal term), in the same class with sane persons, and expect him, under circumstances of great irritation and provocation, to act as the law would require him to act if he were in possession of a sane mind, and a healthy control over his passions.

A man was tried many years back for murdering a stranger whom he accidentally met in a country lane, because he refused, when asked, to give him twopence (that being the sum of money he begged for, at a time when he was proved to be suffering acutely from the pangs of hunger). This man was found guilty and executed. I was at the time much interested in his case, for the evidence of his state of mental disorder (previously to the commission of the murder) was, to my mind, strong and conclusive. It occurred to me that his conduct was quite inconsistent with the hypothesis of sanity—that no man in possession of his reason would have been driven to so horrible an extremity by so trifling a provocation. I, with others, ineffectually interceded with the Secretary of State in his behalf, and endeavoured to save him from the gallows.

As a principle, it may be laid down that a man in a sane state of mind is in a condition to weigh the legal consequences of a suggested criminal line of conduct; there is generally a healthy correspondence between the offence and the action springing out of it.

Before I proceed to the consideration of Homicidal Monomania, and to those morbid states of alleged criminal irresponsibility connected with what are termed blind and irresistible impulses, I would premise that I have always taken exception to these phrases; I think they are unfortunate and unhappy nosological designations of admitted and accepted states of mental disorder associated with a desire to destroy life.

The terms "homicidal monomania," "blind and irresistible impulse," are, I admit, open to grave objections, and to serious abuse. Of the existence of a type of insanity without delirium or apparent delusion, suddenly manifesting itself, and impelling its miserable victims to destroy those nearest and dearest to them, there cannot be a question. There are other cases (and such will be found in most lunatic asylums) in which the mind of the patient appears to be absorbed with one horrible homicidal idea, that being the predominant and characteristic symptom of the mental alienation. A case is recorded in a French journal of a man whose state of mind was made the subject of judicial investigation in France, who for twenty-six years was haunted by an intense desire to destroy human life. He freely confessed that his mind had for this long period been absorbed in this *one* idea.

The Report of the official authorities declared that this man appeared in other respects of sound mind. I subjoin the official account of this remarkable case:—

"I, the undersigned, William Calmeilles, health officer, residing in the principal town of the Canton of Cazals (Lot), certify to all whom it may concern, that, upon the requisition of the mayor of the commune of Marminiat, I have this day been to the village of Brunet, in the aforesaid commune of Marminiat, to decide upon the mental condition of a person named John Glenadel, a husbandman, dwelling in the said village of Brunet.

"I found Glenadel sitting upon his bed, having a cord around his neck, fastened by the other end to the head of the bed; his arms were also tied together at the wrist with another cord. In giving my Report, I do not believe that it can be better made than by recording the conversation which took place between Glenadel and myself, in the presence of his brother and sister-in-law.

"*Question.* Are you unwell?

"*Answer.* I am very well; my health is excellent.

"*Q.* What is your name?

"*A.* John Glenadel.

"*Q.* What is your age?

"*A.* I am forty-three; I was born in '96; see if this is not correct.

"*Q.* Is it by compulsion or by your own consent that you are bound in this manner?

"*A.* It is not only by my consent, but I demanded that it should be done.

"*Q.* Why is this?

"*A.* To restrain me from committing a crime of which I have the greatest horror, and which, in spite of myself, I am constantly impelled to execute.

"*Q.* What is this crime?

"*A.* I have one thought which constantly torments me, and which I cannot conquer—that I must kill my sister-in-law; and I should do it were I not restrained.

"*Q.* How long have you had this idea?

"*A.* About six or seven years.

"*Q.* Have you any cause of complaint against your sister-in-law?

"*A.* Not the least, monsieur; it is only this one unfortunate idea which troubles me, and I feel that I must put it in execution.

"*Q.* Have you ever thought of killing any one besides your sister-in-law?

"*A.* I at first thought of killing my mother; this thought seized me when I was fifteen or sixteen years old, at the age of puberty, in 1812, as I well recollect. Since that time I have not passed one happy hour; I have been the most miserable of men.

"*Q.* Did you conquer this unfortunate idea?

"*A.* In 1822, I could no longer resist, I being at that time twenty-five or six years of age; and to remove this unfortunate inclination, I joined the army in the capacity of a substitute. I was two years in Spain with my regiment, and then returned to France, but this fixed idea followed me everywhere; more than once I was tempted to desert, to go and kill my mother. In 1826 they gave me an unlimited furlough, although it was unsolicited by me, and I returned to my father's house, my fatal idea returning with me. I passed four years with my mother, always having an almost irresistible inclination to kill her.

"*Q.* What did you do then?

"*A.* Then, monsieur, seeing that I should inevitably commit a crime which terrified me and filled me with horror, I, in 1830, rejoined the army, that I might not succumb to this temptation. I left for the second time my father's house, but my fixed idea again followed me, and at last I almost decided to desert, that I might go and kill my mother.

"*Q.* Did you have any cause of complaint against your mother?

"*A.* No, monsieur, I loved her very much; thus, before starting, I said to myself, 'Shall I kill that mother who has exercised so much care over me during my infancy, and who has loved me so well, although I have entertained this fatal thought against her? I will not do it; but I must kill some one.' It was then that the thought of killing my sister-in-law first occurred to me; I have a distinct recollection of this, I being at that time in Dax, and it was in the year 1832. It was then announced to me that my sister-in-law was dead, which was a mistake, it being another relative who had died. I then accepted of the furlough they had offered me, which I should by no means have done had I known that my sister-in-law was still living. When I reached my home, and was informed that she was not dead, I experienced such a sinking and depression of spirits that I became quite sick, and my idea resumed its course.

"*Q.* What instrument do you choose with which to kill your sister-in-law?

"Here Glenadel was much affected; his eyes were bathed in tears; and looking towards his sister-in-law, he replied—'That instrument which would inflict the least pain! But however that may be, the time approaches, I perceive, when she must die, and this is as certain as that God lives.'

"*Q.* Do you not dread to inflict so much misery and anguish upon your brother and your little nephews?

"*A.* The thought of this has troubled me somewhat, but I should receive the punishment due to my crime, and should neither see nor know anything of their affliction; the world would rid itself of a monster such as me, and I should cease to live. I should not expect after this to see a single hour of happiness.

"It here occurred to me that M. Grandsault, of Salviat, my companion and friend, who is at present in Paris, had told me, about a year before, of a young man who, some years previously, had come, accompanied by his mother, to consult him as to his own case, which presented many features very similar to those exhibited by Glenadel. As these cases are so very uncommon, I thought that, perhaps, this person and Glenadel might prove to be the same. I therefore asked him if it was he who had consulted my friend, and he replied in the affirmative.

"*Q.* What did M. Grandsault counsel you?

"*A.* He gave me most valuable advice, and he also bled me.

"*Q.* Did you experience any benefit from this bleeding?

"*A.* Not the least; my unfortunate idea pursued me with the same force.

"*Q.* I am about to make a Report upon your mental condition, from which will be decided whether you shall be placed in an hospital where you may recover from your insanity.

"*A.* My recovery is impossible; but make your Report as quick as possible—time presses. I can control myself but a little longer.

" *Q.* It must be that your parents have instilled into your mind correct moral principles, that they have set before you good examples, and that you yourself have possessed a virtuous mind, to have resisted so long a time this terrible temptation. Here Glenadel was again much affected; he shed tears, and replied, 'You are correct in this, monsieur; but this resistance is more painful than death. I know that I can resist but little longer, and I shall kill my sister-in-law unless I am restrained, as sure as there is a God.'

" 'Glenadel,' said I to him, 'before leaving you, let me ask of you one favour: resist still some days longer, and you shall not see your sister-in-law for a long time, as we will so arrange matters that you can leave here, since you so much desire it.'

" 'Monsieur, I thank you, and I will make arrangement to comply with your recommendation.'

" I left the house, and as I was about to mount my horse, Glenadel called me back, and when I had approached near to him, he said to me, 'Tell these gentlemen that I beseech them to put me in some place from whence it will be impossible for me to escape, for I should make attempts to do so; and were I to succeed in getting away, my sister-in-law would have to die, for I could not avoid killing her; tell these gentlemen that it is my own self who has said this to you.' I assured him that I would do this; but as I saw that he was in a state of great excitement, I asked him if the cord which bound his arms was strong enough, and if he did not think that by a strong effort he could break it. He made an attempt, and then said, 'I fear that I might.' 'But if I should procure for you something that would confine your arms still more securely, would you accept of it?' 'With thanks, monsieur.' 'Then I will ask the commander of the *gendarmes* to give me that with which he is accustomed to confine the arms of prisoners, and I will send it to you.' 'You will confer upon me a great favour.'

'I purposed to make many visits to Glenadel, so as to entirely satisfy myself as to his mental condition; but after the long and painful conversation which I held with him, after what my friend M. Grandsault had told me, after what has been said to me by the brother and sister-in-law of Glenadel, who are so much afflicted at the sad condition of their unfortunate brother, I became well convinced, without farther observation, that John Glenadel was affected with that form of insanity called monomania, characterized in his case by an irresistible inclination to murder—the monomania with which Papavoine and others, fortunately but a small number, were affected.

" Signed at Brunet, in the commune of Marminiat.

"May 21, 1839." " CALMEILLES, *Health Officer.*

Catherine Zeigler was tried at Vienna for the murder of her bastard child. She confessed the act, and said she could not possibly help it; she was forced to do it; she could not resist the desire to commit the murder. The frankness of this her confession, connected with her good character, induced the tribunal to pass a merciful sentence; and on the ground of insanity (which she did not herself plead), she was acquitted, and at length released from prison. But she told the Court, that if they let her escape, they would be responsible for the next murder she committed, for that if ever she had a child again she would certainly kill it. And so, in fact, she did. About ten months after her release from prison, she was delivered of a child, which she soon murdered.

Brought again to her trial, she repeated her old story, and added that she became pregnant merely for the sake of having a child to kill. She was executed for this second murder.

A female was admitted a few years back into the Royal Edinburgh Lunatic Asylum; she had no appreciable disorder of the intellectual powers; she laboured under no delusions. She had a simple abstract desire to kill, or rather, for it took a specific form, to strangle. She made repeated attempts to effect her purpose, attacking every person who came near her, even her own relatives. It appeared to be a matter of indifference to her whom she strangled, so that she succeeded in killing some one. She recovered, under strict discipline, so much self-control as to be permitted to work in the washing-house and laundry; but she still continued to assert that "she must do it;" that "she was certain she would do it some day;" that "she could not help it;" that "surely no one had ever suffered as she had done;" "was not hers an awful case?" And approaching any one, she would gently bring her hand near their throat, and say, mildly and persuasively, "I would just like to do it." She frequently expressed a wish that all the men and women in the world had only one neck, that she might strangle it. Yet this female had a kind and amiable disposition; was beloved by her fellow-patients; so much so, that one of them insisted on sleeping with her, although she herself declared that she was afraid she would not be able to resist the impulse to get up during the night and strangle her. She had been a very religious woman, exemplary in her conduct, very fond of attending prayer meetings and of visiting the sick, praying with them, and reading the Scriptures, or repeating to them the sermons she had heard. It was her second attack of insanity. During the former she had attempted suicide.

The disease was hereditary, and it may be believed that she was strongly predisposed to morbid impulses of this character, when it is stated that her sister and mother both committed suicide. There could be no doubt as to the sincerity of her morbid desires. She was brought to the institution under very severe restraint, and the parties who brought her were under great alarm upon the restraint being removed. After its removal, she made repeated and very determined attacks upon the other patients, the attendants, and the officers of the asylum, and was only brought to exercise sufficient self-control by a system of rigid discipline.

This female was perfectly aware that her impulses were wrong, and that if she had committed any act of violence under their influence, she would have been exposed to punishment. She deplored in piteous terms the horrible propensity.

A few years ago, a gentleman presented himself at a metropolitan lunatic asylum, and begged that he might be received as a patient. He stated that he had just left his solicitor, from whom he, in fact, brought a letter of introduction confirming his account of himself, and that it was necessary he should be placed under some form of restraint, for he had an irresistible desire to murder his wife or one of his children. He then added, that the preceding day he was walking in his garden, when he saw his wife and little girl approaching towards him. His eye at the same moment caught the sight of a hatchet lying on the gravel-walk, and he described that he had the greatest struggle within himself to escape out of the garden before he seized it to strike, perhaps fatally, one or other of them.

He loved his wife and child, he affirmed, dearly; but the homicidal idea haunted him continually, and he felt that he could not trust himself alone in their presence. It should be added, that the last night he slept at home, he did attempt in the middle of the night to strangle his wife, and would have succeeded had not her cries in the scuffle brought in timely assistance. In the midst of all this, during the explanation he gave of his case, he expressed himself well and rationally. His intellect appeared to be unclouded; and it turned out that he was at the same time in communication with his solicitor respecting some proceedings in the Court of Chancery, upon which he gave perfectly sane instructions. I will cite but one additional illustration of this type of insanity. The lunatic in question murdered his wife, and afterwards became a criminal inmate of the State Lunatic Asylum of Massachusetts. He gave the following account of his crime. On the morning of the murder the man was sitting with his wife. He was in a state of excitement; and in these circumstances the noise of the children always disturbed him. In order to render all quiet, the children were sent into a field to play or labour; he and his wife sat by the fire—he on one side, indulging in the gloomiest forebodings; she at work on the other side, doing all in her power to console and comfort him.

After a while she arose, went to the cupboard and poured some wine into a tumbler, brought it to him, and said, in the most cheerful manner, "Come, let us drink and forget our sorrow, and remember our poverty no more." She tasted the wine, and handed it to him, and he drank, and said, in reply, "*I wish it might kill*

*me,*" or, " I might die." She took her seat again by the fire, and went to her work; he arose soon after, without any particular object or design, and walked into an adjoining room. In a moment, the idea of Samson and the weaver's beam rushed into his mind; he instantly seized a weapon which was before him, stepped behind his wife, and gave her the fatal blow. The man, during his confinement, often spoke of the amiable disposition of his wife; he declared that he had no fancied direction from higher powers, and that the thought of killing her never entered his mind until that impulse came upon him, and that it was as sudden as possible, and wholly irresistible. He also spoke of his having made many attempts to commit suicide.

When speaking of insane " irresistible impulses," Mr. Stephen remarks :—

" If the law is to rest satisfied with proof not of an *irresistible*, but merely of an *unresisted* impulse, it gives a sanction to all sorts of crime."

In many conditions of disordered brain and mind, the patient suffers acutely from these "resisted" impulses and morbid mental suggestions. This is one of the most distressing types of nervous and mind disorder coming within the range of the physician's observation and treatment. In many cases, the unhappy patient is fully and painfully conscious of his morbid condition of thought; and it occasionally happens, that so acute is the agony of mind consequent upon the struggle to conquer these suggestions, that relief is sought for in suicide. In this stage of consciousness the patient is occasionally able to appreciate that his sensations are perverted, his thoughts morbid, perceptions false, and his impulses wrongly directed.

Dr. Rush refers to the case of a lady, who prayed fervently that she might be relieved from the horror of her own morbid thoughts by a complete loss of reason!

This terrible consciousness of the approach of insanity, and of the actual existence of the malady, is one of the saddest features in this mysterious disease. The fact has not escaped the wonderful penetration of our great dramatic poet. When Gloster is suffering from profound grief, consequent upon his recognition of Lear's insanity, he exclaims, in the bitterness of his wild despair :—

" The king is mad—how stiff is my vile sense,
That I stand up, and have ingenious feeling
Of my huge sorrows! *Better I were distract;*
*So should my thoughts be severed from my griefs,*
*And woes by wrong imaginations lose*
*The knowledge of themselves.*"

"Such a state as mine," writes a patient, "you are probably unacquainted with, notwithstanding all your experience. I am not conscious of the suspension or decay of any of the powers of my mind. I am as well able as ever I was to attend to my business; my family suppose me in health, yet the horrors of a madhouse are staring me in the face. I am a martyr to a species of persecution from within, which is becoming intolerable. I am urged to say the most shocking things—blasphemous and obscene words are ever on the tip of my tongue. Hitherto, thank God! I have been enabled to resist; but I often think I must yield at last, and then I shall be disgraced for ever, and ruined. I solemnly assure you that I hear a voice which seems to be within me prompting me to utter what I would turn from with disgust if uttered by another. If I were not afraid that you would smile, I should say there is no way of accounting for these extraordinary articulate whisperings, but by supposing that an evil spirit has obtained possession of me for the time; my state is so wretched, that, compared with what I suffer, pain or sickness would appear but trifling evils." *

All crime is alleged to spring from an unresisted and uncontrolled impulse; and a distinguished judge once declared from the bench, when reference was made to the subject of morbid irresistible impulses, that it was one of the objects of punishment to teach men, viciously and criminally disposed, the duty and necessity of restraining their wicked inclinations and impulses. No one doubts the correctness of this principle. But surely it is unphilosophic not to draw a right distinction between a *normal* and *healthy* disposition to crime, and those occasionally resisted and often unhappily irresistible tendencies to what the law considers wicked, vicious, criminal, and punishable acts, clearly connected with, and originating in, a *pathological* condition of the material instrument of thought disordering the mental operations. Was not this distinction entirely lost sight of when Lord Hale committed himself to the dogma that "all crime was partial insanity?" and did not a non-recognition of this great principle lead Dr. Haslam to declare that no mind was sound except that of the Deity? There are *insane* impulses, and *healthy* impulses, to crime and vice; and I think no person acquainted with the phenomena of diseased mind would confound one condition with the other.

A person may, with the object of obtaining some great pecuniary compensation, set fire to his house; another man, with no possible chance or hope of advantage or gain, does the same thing under the influence of an insane impulse. A mother murders her child, to destroy all evidence of her moral delinquency; another mother sacrifices the life of her offspring, to which she is tenderly attached, under the terrible dominion of

* "Essays on Partial Derangement of the Mind in Supposed Connection with Religion," by the late John Cheyne, M.D., pp. 64-5.

a morbid desire to destroy.* A person in a drunken brawl quarrels with his wife because she refuses to supply him with intoxicating drink, and ends by destroying her life; another man, he may be a devoted, affectionate, and loving husband, without exhibiting any previous evidence of insanity, being seized with an attack of homicidal frenzy, rushes upon his wife and cuts her throat! A man may enter a shop, and purloin some article of value; another person, moving in good society, and of high and unimpeachable integrity and above want, may, in a state of mental disorder, commit a similar offence, conscious at the time of the certainty of detection, disgrace, ruin, and punishment. One man practises his profession as a thief—it is his vocation; the other person commits a motiveless crime under the influence of a morbid, insane, and irresistible impulse. I readily admit that such cases require to be most jealously scrutinised. I do not, however, think there can be much practical difficulty in diagnosing and discriminating judicially between the two classes of cases.

To revert, however, to the subject of resisted insane impulses.

Patients often complain of being subject to this type of mental disorder, and feel the necessity for restraint and medical treatment. The suggestion to self-destruction and commission of homicide, without any other evidence of insanity in the popular acceptation of the term, is a common symptom of disorder of the brain and nervous system. The patient, in describing his mental state to his physician, says that the suggestion is "cut your throat,"—"poison—drown yourself,"—"cut your wife's throat,"—"murder your child,"—"poison him." Persons in this state of mind (notwithstanding the presence of great disturbance of the functions of the brain, and disorder of the general health) are able to resist, for a period, these insane suggestions and impulses; but if they should yield to them, and the suggestion be an *irresistible* instead of a *resisted* one, what would Mr. Stephen's opinion be of their legal responsibility in relation to any offence they might commit?

A lady of strong devotional feelings, subject to great nervous disorder, could not repeat the Lord's Prayer without being compelled from within (as she described it) to say, "Our Father, which art in HELL." She could not say "Heaven," although she tried to do so. This poor lady (whose mind was strongly imbued with religious sentiments) suffered great agony of mind in consequence of this horrible suggestion.

I was acquainted with a gentleman—a man of great accomplishments, of high order of intellect, of known literary reputa-

* An occasional occurrence in puerperal insanity.

tion, and of great personal worth—whose mind was for years tortured with morbid suggestions to utter obscene and blasphemous expressions. He eventually destroyed himself; and in a letter which he wrote to me a few days before committing suicide, and which did not reach me until after his death, he said his life was embittered and made wretched by these terrible suggestions; but he thanked God that he had never once yielded to them, and that, although he was a Christian in principle, he felt he was not sinning against God by committing self-destruction, with the object of effectually destroying all chance of his giving utterance to thoughts that might contaminate the minds and morals of others! This was a case of *resisted* suggestion, as far as the thoughts were concerned.

At the Norwich Assizes, in the summer of 1805, Thomas Callaby was tried for the murder of his grandchild. A witness found the prisoner sitting at the side of his bed, one morning in March, about four o'clock: he had dreadfully wounded his wife in different parts of her body. The prisoner's daughter brought down the child with its throat cut; the bloody knife was in the room, and he was charged with, and confessed his crimes, but said, "I do not care anything about it; my wife has heard me say a short time before that *I should certainly murder some one, and I begged to be confined.*" It further appeared in evidence, that he knew when his paroxysms were coming on; and on these occasions he had been known to tie himself down to the floor!

This affords a good illustration of a *resisted*, eventually becoming an *irresistible* impulse; but was not this wretched man as insane when he tied himself down to the floor, and requested his wife to place him in confinement, as when he yielded to the impulse and cut the throat of his grandchild?

Time will not admit of my considering the last division of my subject—namely, those mixed cases of passion, crime, and insanity, associated with a certain diseased temperament and hereditary tendency to mental disease, which, to my mind, clearly justify the merciful consideration of the Court, and some modification of punishment. Take for illustration the case of Lord Ferrers. The crime in this case is said to have been the result of deep-seated revenge. But what was his proved state of mind antecedent to the murder? It was established at his trial that he had long been the subject of *unfounded suspicions of plots and conspiracies, ravings, sudden attacks of fury, denunciations of unprovoked revenge, frantic and insane gesticulations;* that he was in the habit of standing before a glass, spitting and shaking his fist at his reflected image. Lunacy was hereditary in the family, and affected several of his relations. *A solicitor of repu-*

*tation renounced his business on the full persuasion of his being disordered in his brain.* And *long before* the murder of his steward, his nearest relations had deliberated on the expediency of taking out a commission of lunacy against him. *Previously to his separation* from Lady Ferrers, his violence of disposition was so conspicuous, that one of the peers declared from his seat in the House of Lords *that he looked upon him as a maniac, and that if some effectual step was not taken to divest him of the power of doing mischief, he did not doubt but that they should have occasion to try him for murder.* After he shot Mr. Johnson, Lord Ferrers appeared to be conscious of his crime, and showed symptoms of pity; but when the surgeon had dressed the wound, the Earl declared to Mr. Johnson's daughter, as well as to the surgeon, that he intended to kill him, *and did not repent what he had done,* for Johnson was a villain who deserved his fate. He then drank to intoxication, when his hatred became so excited, that he said "*he would not allow the wounded man to be removed to his own house; that he would keep him near himself in order to plague the villain.*" He then retired to his room, *abused and insulted Mr. Johnson, and threatened to shoot him through the head, and was with difficulty restrained from acts of violence.* Even at the moment of death, Lord Ferrers gave evidence of a questionable state of mind. It is recorded that he proceeded to Tyburn in his own carriage drawn by six horses, *dressed gaily for the occasion in a light-coloured suit of clothes embroidered with silver;* and addressing himself to the sheriff, who appeared struck at his singular costume, Lord Ferrers remarked, "You may perhaps think it strange to see me in this dress; but I have my particular reasons for it." Although displeased at being hanged like a common felon, he behaved with propriety and composure, and took an opportunity of declaring he had *no malice* against Mr. Johnson, and that the *murder was committed in a perturbation of mind, occasioned by a variety of crosses and vexations,* but stoutly disclaimed being insane, having had recourse to this plea solely to satisfy his friends. Was not this a case of doubtful sanity, and one of modified responsibility? And would not the claims of justice have been satisfied if Lord Ferrers had been subjected to the severest punishment the law could inflict short of actual death upon the scaffold?

Analogous cases are occasionally recognised in our courts of law, and are acquitted of the capital offence, even when no marked symptoms of mental aberration are proved to have existed.

Mallandine was tried on the charge of attempting to murder her son. She was an unmarried woman, twenty-eight years of age; the child was a boy of six or seven. She was seen to throw him into the Regent's Canal at Haggerstone; and she would have plunged in herself, but a passenger came up and prevented her. The boy was rescued, and she was detained. She then proved to be in a state of wild excitement, brought on by distress. Her counsel, Mr. Cooper, suggested to the jury that the evidence disclosed such a state of mind as did not amount to actual insanity, but prevented her from being aware of the effect of what she was doing. On that argument, apparently, the jury pronounced a verdict of acquittal.

Some years back, a man named Harrison was tried for murder in Scotland, respecting whom the following facts were established:—"He had a wish to join the sect of Quakers, and attended the meetings of that persuasion for some months, where he paid no attention to the worship, but muttered to himself, smelt his Bible, and pricked himself with pins or needles until he lost a considerable quantity of blood. On one occasion he demanded instant admission to the society. He went more than once to the meeting-house early in the morning, and was seen to kneel, and heard to invoke the Virgin Mary, while he wounded himself over with both hands, and smeared the doors with his blood. He habitually wounded his hands, wrists, and arms with needles or pins. He was in the habit of sucking the blood from his own wrists after every two or three mouthfuls of food." Many attempts were made to convince the authorities that these were not the manifestations of a perfectly healthy mind; but they were disregarded, and the poor wretch underwent the penalty of the law.

Much discussion arose at the time of Weston's acquittal for the murder of Mr. Waugh, in Bedford-row. It was questioned whether the verdict of Not Guilty "on the ground of his predisposition to insanity" met the justice of the case. His life was, however, saved. Some months after his trial, his insanity became so well marked that the authorities of Newgate obtained an order from the Secretary of State for his removal to Bethlehem, where I saw and conversed with him in an unmistakable condition of insanity.

When speaking of these modified cases of responsibility, Alison remarks:—

"Cases frequently occur in the highest degree perplexing both to the court and jury, which can only be justly resolved by an application of the principle and mode of proceeding above set forth. They are those in which the accused was to a great degree to blame, but would not probably have committed the fatal act but for some constitutional or supervening derangement which rendered him not *so far responsible* as those who, by enjoying their reason unclouded, had no defence whatever against atrocious actions. In such cases there *is a mixture of guilt and misfortune;* for the former, he should be severely punished; for the latter, the extreme penalty of the law should be remitted."

Has sufficient allowance been made, in the legal consideration of the question of crime committed under the influence of delusion, or irresistible impulse, for a mind prostrated, enfeebled, overpowered, and crushed by a vast and gloomy delusive image, damming up the channels of thought, and destroying all freedom of action?

"I had a species of doubt," says a recovered maniac, describing what his feelings were during his attack; "but no one who has not been deranged can understand how dreadfully true and real a lunatic's insane imaginations appear to him—how slight are his insane doubts."

I may be asked what principle I would propound for the guidance of courts of law in these cases. I cannot but repeat what I have already declared to be my conviction, that in *every criminal case where the question of responsibility arises in the course of judicial inquiry,* IF IT BE POSSIBLE TO ESTABLISH ANY DEGREE OF POSITIVE INSANITY, IT SHOULD ALWAYS BE VIEWED AS A VALID PLEA FOR A CONSIDERABLE MITIGATION OF PUNISHMENT, AND AS PRIMA FACIE EVIDENCE IN FAVOUR OF THE PRISONER; AND IN NO CASE WHERE INSANITY CLEARLY EXISTS (WITHOUT REGARD TO ITS NATURE AND AMOUNT) OUGHT THE EXTREME PENALTY OF THE LAW TO BE INFLICTED.

What, I may be asked, is my test of insanity? I have none. I know of no unerring, infallible, and safe rule or standard, applicable to all cases. The only logical and philosophic mode of procedure in doubtful cases of mental alienation, is to compare the mind of the lunatic at the period of his suspected insanity with its prior natural and healthy condition: in other words, to consider the intellect in relation to itself, and to no artificial *à priori* test. Each individual case must be viewed in its own relations. It is clear that such is the opinion of the judges, notwithstanding they maintained as a test of responsibility a knowledge of right and wrong. Can any other conclusion be drawn from the language used by the judges when propounding in the House of Lords their view of insanity in connexion with crime? "The facts," they say, "of each particular case must of necessity present themselves with *endless variety and with every shade of difference in each case;* and as it is their duty to declare the law upon each particular case, upon facts proved before them, and after hearing arguments of counsel thereon, they deem it at once *impracticable, and at the same time dangerous to the administration of justice, if it were practicable, to attempt to make*

*minute applications of the principles* involved in the answers given by them to the questions proposed." This is a safe, judicious, and philosophic mode of investigating these painful cases; and if strictly adhered to, the ends of justice would be secured, and the requirements of science satisfied.

In considering the question of modified responsibility in connexion with these cases of alleged insanity, we should never lose sight of the fact, that, even if a lunatic be fully exonerated and acquitted in consequence of his state of mind, he is doomed to linger out the remainder of his miserable existence in the criminal wards of a public lunatic asylum.

To talk of a person escaping the extreme penalty of the law on the plea of insanity, as one being subjected to no kind or degree of *punishment,* is a perfect mockery of truth and perversion of language. Suffer no punishment! He is exposed to the severest pain and torture of body and mind that can be inflicted upon a human creature short of being publicly strangled upon the gallows. If the fact be doubted, let a visit be paid to that dreadful *den* at Bethlehem Hospital—

> "*Regions of sorrow, doleful shades, where peace*
> *And rest can never dwell, hope never come,*
> *That comes to all*"—

where the criminal portion of the establishment are confined like wild beasts in an iron cage!

Much has been said of the deterring effects of capital punishment. I do not doubt its having some effect in preventing crime; but I incline to the opinion, that, if the real condition of those confined as criminal lunatics was well understood (assuming the insane to be amenable to the fear of punishment), it would act more potently as a deterring agent than any apprehension they might feel at the prospect of a public execution.

It was the opinion of Beccaria that the impression made by any punishment was in proportion to its *duration,* and not to its *intensity.* "Our sensibility," he observes, "is more readily and permanently affected by slight but reiterated attacks than by a violent but transient affection. For this reason, the putting an offender to death forms a less effectual check to the commission of crimes than the spectacle of a man kept in a state of confinement, and employed in hard labour to make some reparation, by his exertions, for the injury he has inflicted on society."

In judicially estimating cases of crime connected with alleged conditions of insanity, it is our duty always to bear in mind, that, if an error be committed on the side of undue severity, it never can be remedied.

No reparation can be made for so great an injury—for so serious an act of injustice. If a criminal should be unjustly acquitted on the plea of insanity (and I admit such cases have occurred), a degree of injury is undoubtedly done to society, and the confidence in the equitable administration of justice is, to an extent, shaken. But can a judicial mistake like this for one moment be compared with the serious and fatal error of consigning an irresponsible creature to a cruel and ignominious death?

It is well observed by Bentham, that—

"The minimum of punishment is more clearly marked than its maximum. What is *too little* is more clearly observed than what is *too much*. What is not sufficient is easily seen; but it is not possible so exactly to distinguish an excess. An approximation only can be obtained. The irregularities in the force of temptations compel the legislator to increase his punishments until they are not merely sufficient to restrain the ordinary desires of men, but also the violence of their desires when unusually excited. The greatest danger lies in an error on the minimum side, because in this case the punishment is inefficacious; but this error is least likely to occur, a slight degree of attention sufficing for its escape; and when it does exist, it is, at the same time, clear and manifest, and easy to be remedied. An error on the maximum side, on the contrary, is that to which legislators and men in general are naturally inclined—antipathy, or a want of compassion for individuals who are represented as dangerous and vile, pushes them onward to an undue severity. It is on this side, therefore, that we should take the most precautions, as on this side there has been shown the greatest disposition to err."

---

